# Accuracy of diabetes screening methods used for people with tuberculosis, Indonesia, Peru, Romania, South Africa

**DOI:** 10.2471/BLT.17.206227

**Published:** 2018-08-27

**Authors:** Daniel Grint, Bachti Alisjhabana, Cesar Ugarte-Gil, Anca-Leila Riza, Gerhard Walzl, Fiona Pearson, Rovina Ruslami, David A J Moore, Mihai Ioana, Susan McAllister, Katharina Ronacher, Raspati C Koeseomadinata, Sarah R Kerry-Barnard, Jorge Coronel, Stephanus T Malherbe, Hazel M Dockrell, Philip C Hill, Reinout Van Crevel, Julia A Critchley, on behalf of the TANDEM consortium

**Affiliations:** a Tropical Epidemiology Group, London School of Hygiene & Tropical Medicine, London, England.; b Infectious Disease Research Centre, Faculty of Medicine, Universitas Padjadjaran, Bandung, Indonesia.; c Facultad de Medicina Alberto Hurtado, Universidad Peruana Cayetano Heredia, Lima, Peru.; d Department of Internal Medicine and Radboud Center for Infectious Diseases, Radboud University Medical Center, Nijmegen, Netherlands.; e Division of Molecular Biology and Human Genetics, Stellenbosch University, Cape Town, South Africa.; f Population Health Research Institute, St George’s University of London, Cranmer Terrace, London SW17 0RE, England.; g Laboratorio de Investigación y Desarrollo, Universidad Peruana Cayetano Heredia, San Martin de Porres, Peru.; h Human Genomics Laboratory, Universitatea de Medicina si Farmacie din Craiova, Craiova, Romania.; i Centre for International Health, University of Otago, Dunedin, New Zealand.; j Mater Medical Research, Translational Research Institute, University of Queensland, Brisbane, Australia.; k Department of Immunology and Infection, London School of Hygiene & Tropical Medicine, London, England.

## Abstract

**Objective:**

To evaluate the performance of diagnostic tools for diabetes mellitus, including laboratory methods and clinical risk scores, in newly-diagnosed pulmonary tuberculosis patients from four middle-income countries.

**Methods:**

In a multicentre, prospective study, we recruited 2185 patients with pulmonary tuberculosis from sites in Indonesia, Peru, Romania and South Africa from January 2014 to September 2016. Using laboratory-measured glycated haemoglobin (HbA1c) as the gold standard, we measured the diagnostic accuracy of random plasma glucose, point-of-care HbA1c, fasting blood glucose, urine dipstick, published and newly derived diabetes mellitus risk scores and anthropometric measurements. We also analysed combinations of tests, including a two-step test using point-of-care HbA1cwhen initial random plasma glucose was ≥ 6.1 mmol/L.

**Findings:**

The overall crude prevalence of diabetes mellitus among newly diagnosed tuberculosis patients was 283/2185 (13.0%; 95% confidence interval, CI: 11.6–14.4). The marker with the best diagnostic accuracy was point-of-care HbA1c (area under receiver operating characteristic curve: 0.81; 95% CI: 0.75–0.86). A risk score derived using age, point-of-care HbA1c and random plasma glucose had the best overall diagnostic accuracy (area under curve: 0.85; 95% CI: 0.81–0.90). There was substantial heterogeneity between sites for all markers, but the two-step combination test performed well in Indonesia and Peru.

**Conclusion:**

Random plasma glucose followed by point-of-care HbA1c testing can accurately diagnose diabetes in tuberculosis patients, particularly those with substantial hyperglycaemia, while reducing the need for more expensive point-of-care HbA1c testing. Risk scores with or without biochemical data may be useful but require validation.

## Résumé

**Objectif:**

Évaluer la performance des outils de diagnostic du diabète sucré, et notamment les méthodes de laboratoire et les cotes de risque clinique, chez les patients souffrant d'une tuberculose pulmonaire récemment diagnostiquée dans quatre pays à revenu intermédiaire.

**Méthodes:**

Dans le cadre d'une étude multicentrique et prospective, nous avons recruté 2185 patients atteints de tuberculose pulmonaire dans différents endroits en Afrique du Sud, en Indonésie, au Pérou et en Roumanie entre janvier 2014 et septembre 2016. En utilisant comme référence l'hémoglobine glyquée (HbA1c) mesurée en laboratoire, nous avons évalué la précision diagnostique de la glycémie aléatoire, de l'HbA1c sur le lieu d'intervention, de la glycémie à jeun, de la bandelette urinaire, des cotes de risque déjà publiées et récemment obtenues pour le diabète sucré et des mesures anthropométriques. Nous avons également analysé des combinaisons de tests, et notamment un test en deux étapes qui utilise l'HbA1c sur le lieu d'intervention lorsque la glycémie aléatoire initiale est ≥ 6,1 mmol/L.

**Résultats:**

La prévalence brute générale du diabète sucré chez les patients souffrant d'une tuberculose récemment diagnostiquée était de 283/2185 (13,0%; intervalle de confiance, IC, à 95%: 11,6–14,4). Le marqueur fournissant la meilleure précision diagnostique était l'HbA1c sur le lieu d'intervention (aire sous la courbe ROC: 0,81; IC à 95%: 0,75–0,86). La meilleure précision diagnostique générale a été obtenue à l'aide d'une cote de risque calculée au moyen de l'âge, de l'HbA1c sur le lieu d'intervention et de la glycémie aléatoire (aire sous la courbe ROC: 0,85; IC à 95%: 0,81–0,90). Une forte hétérogénéité entre les différents endroits a été constatée pour tous les marqueurs, mais le test en deux étapes a donné de bons résultats en Indonésie et au Pérou.

**Conclusion:**

L'utilisation de la glycémie aléatoire suivie de l'HbA1c sur le lieu d'intervention permet de diagnostiquer avec précision le diabète chez les patients atteints de tuberculose, en particulier lorsqu'ils souffrent d'une importante hyperglycémie, tout en diminuant le besoin de tests HbA1c sur le lieu d'intervention, plus coûteux. Les cotes de risque associées ou non à des données biochimiques peuvent être utiles, mais doivent être validées.****

## Resumen

**Objetivo:**

Evaluar el rendimiento de las herramientas de diagnóstico para la diabetes mellitus, incluidos los métodos de laboratorio y los índices de riesgo clínico, en pacientes recién diagnosticados con tuberculosis pulmonar de cuatro países de ingresos medios.

**Métodos:**

En un estudio prospectivo multicéntrico, se seleccionaron 2185 pacientes con tuberculosis pulmonar de algunas regiones de Indonesia, Perú, Rumanía y Sudáfrica entre enero de 2014 y septiembre de 2016. Usando hemoglobina glicosilada medida en laboratorio (HbA1c) como el patrón de excelencia, se midió la precisión diagnóstica de glucosa plasmática al azar, estando HbA1c en el punto de atención, glucemia en sangre en ayunas, tira reactiva de orina, índices de riesgo de diabetes mellitus publicados recientemente y medidas antropométricas. También se analizaron combinaciones de pruebas, incluida una prueba de dos pasos con HbA1c en el punto de atención cuando la glucosa plasmática aleatoria inicial fue ≥ 6,1mmol/L.

**Resultados:**

La prevalencia general cruda de diabetes mellitus entre los pacientes con tuberculosis recién diagnosticados fue de 283/2185 (13,0 %, intervalo de confianza del 95 %, IC: 11,6–14,4). El marcador con la mejor precisión diagnóstica fue la HbA1c en el punto de atención (área bajo la curva característica operativa del receptor: 0,81; IC del 95 %: 0,75–0,86). Un índice de riesgo derivado de la edad, la HbA1c en el punto de atención y la glucosa plasmática al azar obtuvieron la mejor precisión diagnóstica general (área bajo la curva: 0,85; IC del 95 %: 0,81–0,90). Hubo una heterogeneidad sustancial entre las regiones para todos los marcadores, pero la prueba combinada de dos pasos funcionó de manera correcta en Indonesia y Perú.

**Conclusión:**

La glucosa plasmática al azar seguida de la prueba de HbA1c en el punto de atención puede diagnosticar con precisión la diabetes en pacientes con tuberculosis, particularmente en aquellos con hiperglucemia sustancial, mientras se reduce la necesidad de pruebas más costosas de HbA1c en el punto de atención. Los índices de riesgo con o sin datos bioquímicos pueden ser útiles, pero requieren validación.

## ملخص

**الغرض:**

تقييم أداء أدوات تشخيص مرض السكري، بما في ذلك الطرق المختبرية ودرجات المخاطر السريرية، لدى المرضى الذين تم تشخيص إصابتهم حديثاً بالسل الرئوي من أربعة بلدان متوسطة الدخل.

**الطريقة:**

في دراسة استباقية متعددة المراكز، قمنا بالاستعانة بـ 2185 مريضًا بمرض السل الرئوي من مواقع في إندونيسيا وبيرو وجنوب إفريقيا ورومانيا من يناير/كانون ثاني 2014 إلى سبتمبر/أيلول 2016. وعن طريق قياس الهيموغلوبين الغليكوزيلاتي في المختبر (HbA1c) كمعيار أساسي، قمنا بقياس مدى دقة تشخيص كل من جلوكوز البلازما العشوائي، واختبار HbA1c في أماكن الرعاية، وجلوكوز الدم أثناء الصيام، ومقياس البول، ودرجات المخاطر المنشورة والمستنبطة حديثا من داء السكري، والقياسات الأنثروبومترية. قمنا أيضا بتحليل مجموعات من الاختبارات، بما في ذلك اختبار من خطوتين باستخدام اختبار HbA1c في أماكن الرعاية عندما كان مستوى جلوكوز البلازما العشوائي أكبر من أو يساوي 6.1 مليمول/ لتر.

**النتائج:**

كان معدل الانتشار الكلي لمرض السكري بين المرضى الذين تم تشخيص إصابتهم حديثًا بالسل هو 283/2185 (13.0%؛ بفاصل ثقة 95%، فاصل ثقة: 11.6 إلى 14.4).كانت العلامة ذات أفضل دقة تشخيصية هي اختبار HbA1c في أماكن الرعاية (المساحة تحت منحنى خاصية التشغيل للمستقبِل: 0.81؛ بفاصل ثقة 95%: 0.75 إلى 0.86). كانت درجة الخطر المشتقة باستخدام العمر، واختبار HbA1c في أماكن الرعاية، وجلوكوز البلازما العشوائي، هي أفضل دقة تشخيصية عامة (المساحة تحت المنحنى: 0.85؛ بفاصل ثقة 95%: 0.81 إلى 0.90). كانت هناك عدم تجانس واضح بين المواقع لكل العلامات، إلا أن الاختبار المكون من خطوتين قدم أداءً جيدًا في إندونيسيا وبيرو.

**الاستنتاج:**

يمكن لاختبار جلوكوز البلازما العشوائي، ويليه اختبار HbA1c في أماكن الرعاية، تشخيص مرض السكري بدقة في مرضى السل، وخاصة أولئك الذين يعانون من ارتفاع شديد في سكر الدم، مع تقليل الحاجة إلى اختبار HbA1c الأكثر تكلفة في أماكن الرعاية. قد تكون درجات الخطر مفيدة، سواء مع بيانات كيميائية حيوية أو بدونها، ولكنها تتطلب التحقق من الصحة. ****

## 摘要

**目的:**

评估用于四个中等收入国家新诊断结核病患者的糖尿病诊断工具的表现，其中包括实验室方法和临床风险评分。

**方法:**

在这项多中心、前瞻性的研究中，自 2014 年 1 月起至 2016 年 9 月，我们从秘鲁、罗马尼亚、南非和印度尼西亚各地招募了 2185 名肺结核患者。使用实验室测量的糖化血红蛋白 (HbA1c) 为黄金标准，我们测量了随机血糖、糖化血红蛋白 (HbA1c) 床旁检测、空腹血糖、尿液常规检查、已发表的和新导出的糖尿病风险评分以及人体测量法的诊断准确度。我们还对组合检测法进行了分析，其中包括两步检测法：即在初始随机血糖为临界值 ≥6.1 mmol/L 时，进行糖化血红蛋白 (HbA1c) 床旁检测。

**结果:**

新诊断结核病患者中，糖尿病的整体患病率为 283/2185（13.0％；95％ 置信区间，CI：11.6–14.4）。最准确的诊断指标是糖化血红蛋白 (HbA1c) 床旁检测（受试者工作特征曲线下的区域：0.81；95％ 置信区间，CI：0.75-0.86）。使用年龄、糖化血红蛋白 (HbA1c) 床旁检测和随机血糖得出的风险评分整体诊断准确度最佳（受试者工作特征曲线下的区域：0.85；95％ 置信区间，CI：0.81-0.90）。不同地点的指标之间存在显著的异质性，但两步组合检测法在秘鲁和印度尼西亚表现良好。

**结论:**

在随机血糖检测后再进行糖化血红蛋白 (HbA1c) 床旁检测可以准确诊断结核病患者是否患有糖尿病（尤其针对患有严重高血糖的患者），同时减少对更为昂贵的糖化血红蛋白 (HbA1c) 床旁检测的需求。内附或未附生化指标的风险评分可能有用，但仍需验证。****

## Резюме

**Цель:**

Оценить эффективность инструментов диагностики сахарного диабета (включая лабораторные методы и показатели клинического риска) у пациентов с недавно диагностированным туберкулезом легких из четырех стран со средним уровнем доходов.

**Методы:**

С января 2014 года по сентябрь 2016 года авторы провели многоцентровое проспективное исследование, в котором приняли участие 2185 пациентов с туберкулезом легких, проживающих в Индонезии, Перу, Румынии и Южной Африке. Используя измеренный в лаборатории гликозилированный гемоглобин (HbA1c) в качестве общепринятого стандарта, авторы измерили диагностическую точность выборочного определения уровня глюкозы в плазме; уровня HbA1c, определяемого в месте предоставления медицинской помощи; уровня глюкозы в крови натощак и определения сахара в моче с помощью индикаторной полоски с учетом опубликованных и недавно полученных оценок риска заболевания сахарным диабетом и антропометрических измерений. Мы также проанализировали комбинации тестов, в том числе двухэтапный тест с использованием HbA1c, измеренного в месте предоставления медицинской помощи, при начальном случайном уровне глюкозы в плазме ≥ 6,1 ммоль/л.

**Результаты:**

Общая распространенность сахарного диабета среди пациентов с недавно выявленным туберкулезом составила 283 случая на 2185 человек (13,0%, 95%-й доверительный интервал, ДИ: 11,6–14,4). Маркер с наибольшей диагностической точностью — HbA1c, измеренный в месте предоставления медицинской помощи (область под кривой ROC: 0,81; 95%-й ДИ: 0,75–0,86). Оценка риска в зависимости от возраста, HbA1c, измеренного в месте предоставления медицинской помощи, и выборочного определения глюкозы в плазме имела наилучшую общую диагностическую точность (область под кривой: 0,85; 95%-й ДИ: 0,81–0,90). В различных местах наблюдалась существенная гетерогенность всех маркеров, при этом двухступенчатый комбинированный тест дал хорошие показатели для Индонезии и Перу.

**Вывод:**

Выборочно определяемый уровень глюкозы в плазме с последующим тестированием HbA1c в месте предоставления медицинской помощи может точно диагностировать диабет у пациентов с туберкулезом, особенно у пациентов с существенной гипергликемией, уменьшая необходимость в более дорогостоящем тестировании HbA1c в месте предоставления медицинской помощи. Оценки риска по данным биохимического анализа крови или без них могут быть полезными, но нуждаются в проверке.

## Introduction

Tuberculosis is a major global public health concern. In 2016, the World Health Organization (WHO) estimated that there were 10.4 million new tuberculosis patients worldwide and 1.8 million tuberculosis-related deaths.^1^ Diabetes mellitus affects the host immune response to tuberculosis, and people with diabetes have a threefold increased risk of developing active tuberculosis.^2^ In 2015, the International Diabetes Federation estimated that there were 415 million adults living with diabetes mellitus worldwide, many undiagnosed, and mostly living in low- and middle-income countries where there is often a high burden of tuberculosis.^1^^,^^3^ Diabetes patients with concurrent tuberculosis also have poorer tuberculosis treatment outcomes,^4^^,^^5^ so it is important to identify these patients promptly to optimize treatment.

Current WHO guidelines for diagnosing diabetes mellitus in healthy people at high risk of the disease are based on identifying diabetes symptoms (polyuria, polydipsia, unexplained weight loss), in combination with measurement of fasting plasma glucose, oral glucose tolerance or glycated haemoglobin (HbA1c). Repeated laboratory measurements are needed if the patient does not have symptoms.^4^^,^^6^ WHO recommends screening newly diagnosed tuberculosis patients for diabetes, but has not recommended any specific method.^7^ The symptoms of active tuberculosis disease overlap with those of diabetes, and the inflammation associated with infectious disease can increase insulin resistance, which complicates the diagnosis of diabetes.^8^

Several risk scores have been developed to detect undiagnosed diabetes based on clinical data.^9^ However, the scores are strongly based on anthropometric markers (body mass index, waist–hip ratio) that are affected by the weight loss associated with tuberculosis disease. Previous studies screening tuberculosis patients for undiagnosed diabetes have mainly considered operational issues and feasibility.^9^^–^^12^ Few studies have compared the accuracy of more than one screening test, including simple and affordable tests such as risk scores and some point-of-care tests. Furthermore, studies have not estimated the accuracy of two-step screening processes or risk scores for diabetes diagnosis derived specifically in tuberculosis patients. We therefore aimed to evaluate the performance of diagnostic tools for diabetes mellitus, including standard glucose testing, two-step screening and clinical risk scores, in newly-diagnosed pulmonary tuberculosis patients from four middle-income countries.

## Methods

### Study design

The Concurrent Tuberculosis and Diabetes Mellitus (TANDEM) study aimed to improve the screening and management of combined tuberculosis and diabetes mellitus.^13^ This multicentre, prospective study has field sites in Indonesia, Peru, Romania and South Africa. These are countries with diverse health-care systems and population demographics, but a relatively high burden of tuberculosis and an increasing prevalence of diabetes.^1^^,^^3^ Further information on the study design and methods are available from a data repository.^14^

### Study procedures

At each site we recruited patients with newly diagnosed pulmonary tuberculosis based on symptoms, chest X-rays and sputum culture examination. Consecutive pulmonary tuberculosis patients presenting for treatment at study sites after January 2014 were recruited up until September 2016. We excluded patients from the diagnostic accuracy analysis if they were known to have diabetes (i.e. self-reporting a previous diagnosis of diabetes mellitus from a health-care practitioner or on antidiabetic medication). We used laboratory measurement of HbA1c as the gold standard for the diagnosis of diabetes, with a diagnostic cut-off point ≥ 6.5%.^4^ To account for potential transient hyperglycaemia, we made secondary analyses, defining diabetes by repeated raised HbA1c at the end of tuberculosis treatment with a higher cut-off at baseline (≥ 7.0%)^8^ or by fasting plasma glucose.

We screened for diabetes at the time of recruitment, and patients with initial raised HbA1c were offered a confirmatory test. We aimed to repeat the HbA1c test for all patients at the end of tuberculosis treatment, which was 6 months after recruitment. Other data collected include demographic data, height, weight, family history of diabetes, self-reported gestational diabetes or delivery of a large baby (> 4 kg weight), anthropometric measurements (waist circumference and weight-to-hip ratio), levels of physical activity and consumption of fruits and vegetables. We recorded point-of-care diabetes markers (HbA1c), random plasma glucose and fasting plasma glucose for those with an initial random plasma glucose ≥ 6.1 mmol/L and urinary dipstick for glucose. Point-of-care HbA1c measurements were made using the HemoCue® HbA1c 501 test (HemoCue AB, Ängelholm, Sweden). In Romania, HemoCue® was not available, so we used QuoTest® (EKF Diagnostics, Cardiff, United Kingdom of Great Britain and Northern Ireland). In addition, we sent blood samples for HbA1c testing in an accredited laboratory with certification from the National Institutes of Diabetes and Digestive and Kidney Diseases.^15^ We calculated three previously published diabetes risk scores for each patient: the Finnish diabetes risk score,^16^ the Indian risk score^17^ and the Oman diabetes risk scores.^18^ We selected these scores based on a recent review^9^ due to their higher sensitivity in detecting diabetes, repeatability in validation studies, demographic diversity and complementary variables.

We also evaluated a two-step combination test, which was a random plasma glucose test, followed by a point-of-care HbA1c test in certain circumstances. If the random plasma glucose test result was < 6.1 mmol/L the individual was determined not to have diabetes, and no further testing was performed. For random plasma glucose ≥ 11.1 mmol/L, the individual was determined to have diabetes and no further testing was performed. For random plasma glucose between these values a point-of-care HbA1c test would be performed. This combination was chosen since the initial test is cheap and widely available, and low levels are more likely to rule out diabetes. The second test is more expensive, but used only on those with random plasma glucose ≥ 6.1 mmol/L. Neither test requires the patient to fast, so both can be performed in one clinic visit.

### Statistical methods

In cross-sectional analysis, we compared the diagnostic accuracy of random plasma glucose, fasting plasma glucose, point-of-care HbA1c, urine dipstick and the three published diabetes risk scores and the two-step combination test. We calculated sensitivity, specificity and the area under the receiver operating characteristic (ROC) curve. We defined cut-offs for each diagnostic measure using established values from the published literature,^6^^,^^9^^,^^19^^,^^20^ and two data-driven values, one chosen to provide the maximum combination of sensitivity and specificity, the other a sensitivity of at least 80%. Sample size calculations were carried out for a pre-specified sensitivity and precision.^14^

We developed new risk scores using logistic regression models and backward variable selection. These scores aimed to assess the diagnostic accuracy of combining random plasma glucose and point-of-care HbA1c with anthropometric and risk factor measurements. The variables included were derived from previously published risk scores,^9^ including age, family history of diabetes, physical activity (active for more than 30 minutes per day), daily consumption of fruits and vegetables, taking antihypertensive medications, waist circumference, weight-to-hip ratio, body mass index, random plasma glucose and point-of-care HbA1c. To maximize diagnostic accuracy we also used first- and second-order fractional polynomial methods for the continuous predictors random plasma glucose, point-of-care HbA1c, age and body mass index.^14^ In a separate model for women, we included history of gestational diabetes and delivery of a large baby. The full TANDEM score included all variables retained in the backward selection model, with *P* < 0.1 required to remain in the model. The restricted TANDEM score followed the same method, but omitted point-of-care HbA1c as a covariate due to the cost implications.

We summarized the areas under the ROC curve and sensitivity and specificity estimates over the whole study population. Due to considerable diagnostic heterogeneity, we stratified estimates by country. We also assessed diagnostic accuracy in the sensitivity analyses when defining diabetes using fasting plasma glucose (≥ 7 mmol/L), where fasting plasma glucose was available. Statistical analyses were performed using SAS, version 9.4 (SAS Institute, Cary, United States of America).

## Results

### Baseline characteristics

A total of 2185 patients with newly-diagnosed pulmonary tuberculosis were enrolled: 748 in Indonesia, 600 in Peru, 506 in Romania and 331 in South Africa. Tuberculosis was bacteriologically confirmed in 1867 (85.4%) patients, with the rest being diagnosed clinically. A quarter of patients (538; 24.7%) reported a previous diagnosis of tuberculosis, 363 (64.0%) of whom had completed treatment. At screening, 2032 patients (93.2%) had cough, 1690 (77.8%) had weight loss and 1466 (67.7%) reported night sweats. Among patients agreeing to testing, co-infection with human immunodeficiency virus was found in one patient (0.2%) in Romania, 26 (3.5%) in Indonesia, 23 (3.8%) in Peru and 31 (9.4%) in South Africa.

For the diagnostic accuracy analysis we excluded 183 patients with self-reported diabetes before recruitment and also 63 with no measurement of laboratory HbA1c. Baseline characteristics and diabetes diagnostic test data are summarized in Table 1 for the 1939 newly-diagnosed pulmonary tuberculosis patients with no previous diabetes diagnosis: 649 in Indonesia, 562 in Peru, 469 in Romania and 259 in South Africa. In total, 100 patients were newly diagnosed with diabetes, a crude prevalence of 5.2% (95% CI: 4.2–6.2), ranging from 3.0% (95% CI: 1.8–4.8; 17 patients) in Peru to 6.9% (95% CI: 4.2–10.8; 18 patients) in South Africa. The overall crude prevalence of diabetes mellitus (both new and previously diagnosed) was 13.0% (95% CI: 11.6–14.4; 283 patients).

**Table 1:** Baseline characteristics and diabetes mellitus status of newly-diagnosed pulmonary tuberculosis patients with no previous diabetes mellitus diagnosis in the Concurrent Tuberculosis and Diabetes Mellitus study, 2014–2016

Variable	Indonesia			Peru			Romania			South Africa	
	*n*	Value		*n*	Value		*n*	Value		*n*	Value
Newly diagnosed diabetes mellitus,^a^ no. of patients (%)	649	34 (5.2)		562	17 (3.0)		469	31 (6.6)		259	18 (6.9)
Male sex, no. of patients (%)	649	377 (58.1)		562	328 (58.4)		469	330 (70.7)		259	166 (64.1)
Age, median (IQR) years	649	35.0 (26.0–47.0)		562	28.0 (22.2–40.0)		469	41.0 (28.0–53.0)		259	33.0 (27.0–47.0)
Body mass index, median (IQR) kg/m^2^	646	17.9 (16.1–20.0)		562	22.0 (19.9–24.2)		466	20.2 (18.5–22.0)		258	18.4 (16.8–20.3)
Weight-to-hip ratio, median (IQR)	647	0.8 (0.8–0.9)		562	0.9 (0.8–0.9)		461	0.8 (0.8–0.9)		257	0.8 (0.8–0.9)
Random plasma glucose, median (IQR) mmol/L	649	5.5 (4.9–6.3)		558	5.4 (4.8–6.2)		434	5.8 (5.1–7.4)		258	5.7 (5.0–6.8)
Fasting plasma glucose, median (IQR) mmol/L	120	4.6 (4.1–5.2)		446	4.9 (4.4–5.4)		465	4.9 (4.3–5.6)		0^b^	NA
Point-of-care HbA1c, median (IQR) %	639	5.8 (5.4–6.2)		542	5.8 (5.6–6.3)		409	5.5 (5.2–5.8)		250	5.5 (5.2–5.9)
Finnish diabetes risk score,^c^ median (IQR)	644	1.0 (1.0–3.0)		561	3.0 (1.0–5.0)		453	1.0 (1.0–4.0)		256	3.0 (1.0–5.0)
Indian risk score,^c^ median (IQR)	649	20.0 (0.0–30.0)		562	20.0 (0.0–30.0)		469	20.0 (0.0–30.0)		259	30.0 (0.0–30.0)
Oman risk score,^c^ median (IQR)	649	2.0 (0.0–8.0)		562	2.0 (2.0–9.0)		469	7.0 (2.0–9.0)		259	2.0 (0.0–8.0)
Laboratory-measured glycated haemoglobin, median (IQR) %											
No diabetes mellitus	615	5.5 (5.3–5.8)		545	5.5 (5.2–5.8)		438	5.8 (5.5–6.1)		241	5.8 (5.5–6.0)
Diabetes mellitus	34	10.8 (8.0–12.9)		17	7.9 (6.8–10.6)		31	6.7 (6.6–6.9)		18	6.8 (6.6–7.0)

HbA1c: glycated haemoglobin; IQR: interquartile range; NA: not applicable.

^a^ We defined diabetes mellitus as HbA1c ≥ 6.5%.

^b^ Fasting plasma glucose testing was not done in South Africa. Fasting plasma glucose was intended to be measured at baseline when random plasma glucose was ≥ 6.1 mmol/L.

^c^ Finnish diabetes risk score includes the variables age, body mass index, waist circumference, current blood pressure medication, history of high blood glucose, physical activity and consumption of fruits and vegetables, and takes values 0–20 with a suggested optimal diagnostic cut-off of ≥ 9.15.^16^ Indian risk score includes age, body mass index, waist circumference, family history of diabetes mellitus and physical activity, has values 0–42 with a suggested cut-off of ≥ 21.^17^ Oman diabetes risk score includes age, waist circumference, sex, body mass index, hypertension, and family history of diabetes mellitus and has values 0–25 with a suggested cut-off of ≥ 10.^18^

Notes: Patients were recruited to the study from January 2014 to September 2016. *N* is the total number of patients. Participant numbers reported here may vary slightly from some other TANDEM consortium analyses owing to minor differences in inclusion criteria or recruitment period.

### Diabetes risk scores

Logistic regression parameter estimates and diabetes risk scores are shown in Table 2. In univariate analysis, age, family history of diabetes, physical activity, taking antihypertensive medications, waist circumference, body mass index category, random plasma glucose and point-of-care HbA1c were all associated with incident diabetes (*P* < 0.1). Data on the relationships of age, random plasma glucose and point-of-care HbA1c with the probability of diabetes are available from the repository.^14^

**Table 2:** Univariate and multivariate logistic regression models; development of risk scores in the Concurrent Tuberculosis and Diabetes Mellitus study, 2014–2016

Covariate	Univariate model					Full TANDEM score^a^				Restricted TANDEM score^b^		
	*β*	OR (95% CI)			*β*		*P*		*β*		*P*
Age, per year	0.05	1.06 (1.04–1.07)			NA		NA		NA		NA
Second order fractional polynomial: age^c^											
*β_1_*	0.010	NA^d^			0.0073		0.0039		0.0077		0.0012
*β_2_*	−0.0022	NA^d^			−0.0016		0.0060		−0.0017		0.0021
Sex: male	−0.01	1.0 (0.7–1.5)			NA		NA		NA		NA
Family history of diabetes mellitus: yes	0.55	1.7 (1.0–3.0)			NA		NA		NA		NA
Physical activity < 30 minute/day	0.41	1.5 (0.9–2.5)			NA		NA		0.64		0.040
Fruit and vegetable consumption: daily	0.14	1.2 (0.8–1.8)			NA		NA		NA		NA
Anti-hypertension medication: yes	0.45	1.6 (0.6–4.0)			NA		NA		NA		NA
Waist circumference: male > 94 cm; female > 80 cm	1.01	2.7 (1.7–4.5)			NA		NA		NA		NA
Waist-to-hip ratio: male > 0.90; female > 0.80	0.24	1.3 (0.9–1.9)			NA		NA		NA		NA
Body mass index^e^											
≤ 25 kg/m^2^	Ref.	Ref.			NA		NA		NA		NA
< 30 kg/m^2^	0.86	2.4 (1.3–4.3)			NA		NA		0.36		0.36
≥ 30 kg/m^2^	1.90	6.7 (2.6–17.1)			NA		NA		1.34		0.038
Random plasma glucose,^b^ per mmol/L	0.52	1.7 (1.5–1.8)			NA		NA		NA		NA
Random plasma glucose: *β_1_*	0.030	NA^d^			0.019		< 0.0001		0.025		< 0.0001
Point-of-care HbA1c, per %	0.85	2.4 (2.0–2.7)			NA		NA		NA		NA
Log (point-of-care HbA1c): *β_1_*	6.87	NA^d^			5.04		< 0.0001		NA		NA

CI: confidence interval; HbA1c: glycated haemoglobin; NA: not applicable; OR: odds ratio; Ref. reference category; TANDEM**:** Concurrent Tuberculosis and Diabetes Mellitus study.

^a^ TANDEM scores were derived from multivariate backward selection logistic regression including all covariates significant in univariate models (*P* < 0.15) as candidate variables. The values for the full score ranged from 8.7–33.5 (median 11.3) and the restricted score 1.1–26.1 (median 2.9).

^b^ Restricted risk scores omitted point-of-care HbA1c from the covariates.

^c^ Second order fractional polynomial was calculated as: *β1* Age^2^ + *β2* Age^2^ log(Age).

^d^ Odds ratios were omitted for fractional polynomial parameters; the continuous probability plots are available from authors’ data repository.^14^

^e^ Global body mass index *P*-values: univariate 0.0002; restricted TANDEM score 0.087.

Note: New diabetes mellitus cases were defined using a single HbA1c measurement (≥ 6.5%).

In the full multivariate stepwise regression model the fractional polynomial form of age, random plasma glucose^2^ and log(point-of-care HbA1c) were selected as independent predictors of diabetes. In the restricted model, the fractional polynomial form of age, physical activity, body mass index category and random plasma glucose^2^ were selected as predictors. In a separate model for women, history of gestational diabetes and delivery of a big baby were assessed, although neither approached statistical significance (*P* > 0.2). The parameter estimates from these models were used to construct two new risk score equations (Box 1).

Box 1 **Two new risk scores for diabetes mellitus**

Full TANDEM score = 0.0073(Age)^2^−0.0016(Age)^2^log(Age)+0.019(RPG)^2^+5.04log (point-of-care HbA1c)

Restricted TANDEM score = 0.0077(Age)^2^−0.0017(Age)^2^log(Age)+0.025(RPG)^2^+0.36(if 25 ≤ BMI ≤ 30)+1.34(if BMI > 30)+0.64(if < 30 minutes physical activity/day)

BMI: body mass index; HbA1c: glycated haemoglobin; RPG: random plasma glucose; TANDEM**:** Concurrent Tuberculosis and Diabetes Mellitus study.

### Diagnostic accuracy of markers

**Fig. 1:**
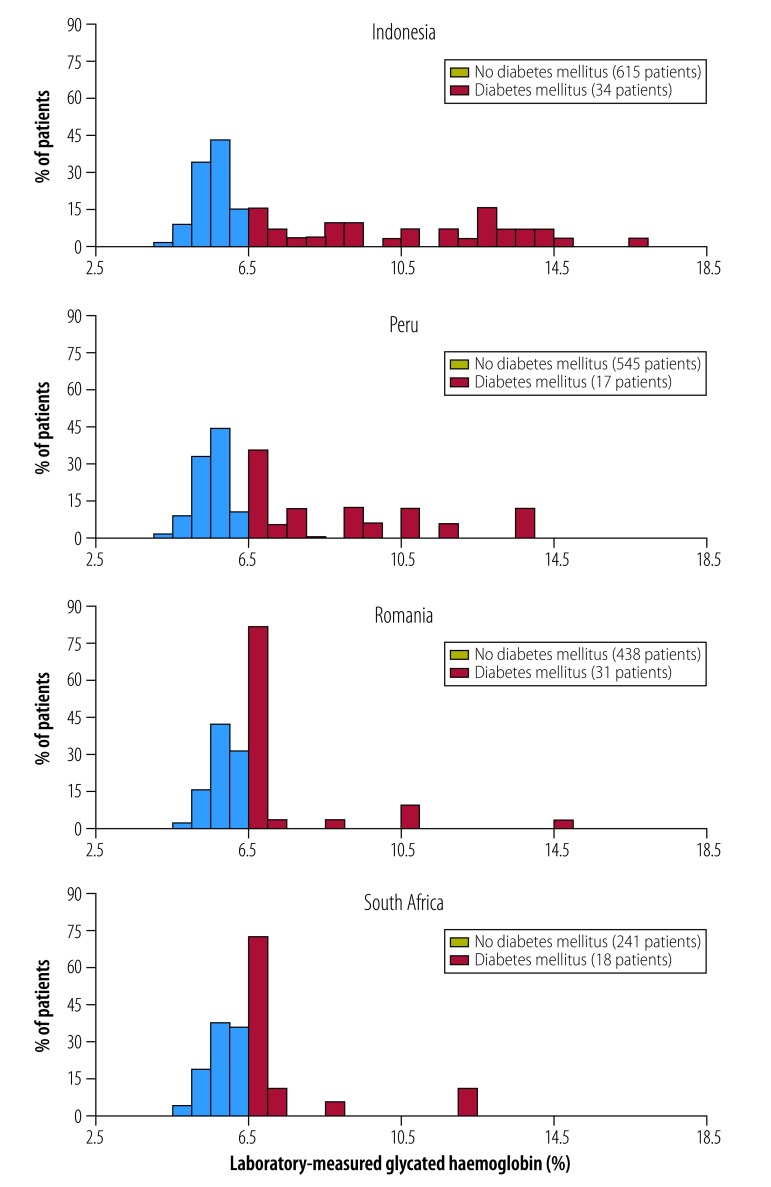
Distribution of laboratory-measured glycated haemoglobin in patients with newly diagnosed tuberculosis recruited to the Concurrent Tuberculosis and Diabetes Mellitus study, by country, 2014–2016

Note: New diabetes mellitus was defined by laboratory-measured glycated haemoglobin ≥ 6.5%.

There was considerable heterogeneity in the degree of laboratory-measured HbA1c associated with diabetes between countries (Fig. 1). In Indonesia and Peru, the median HbA1c level for new diabetes cases was 10.8% (interquartile range, IQR: 8.0–12.9) and 7.9% (IQR: 6.8–10.6), respectively, far above the recognized 6.5% diagnostic cut-off for diabetes. In Romania and South Africa the levels were 6.7% (IQR: 6.6–6.9) and 6.8% (IQR: 6.6–7.0), respectively.

**Fig. 2:**
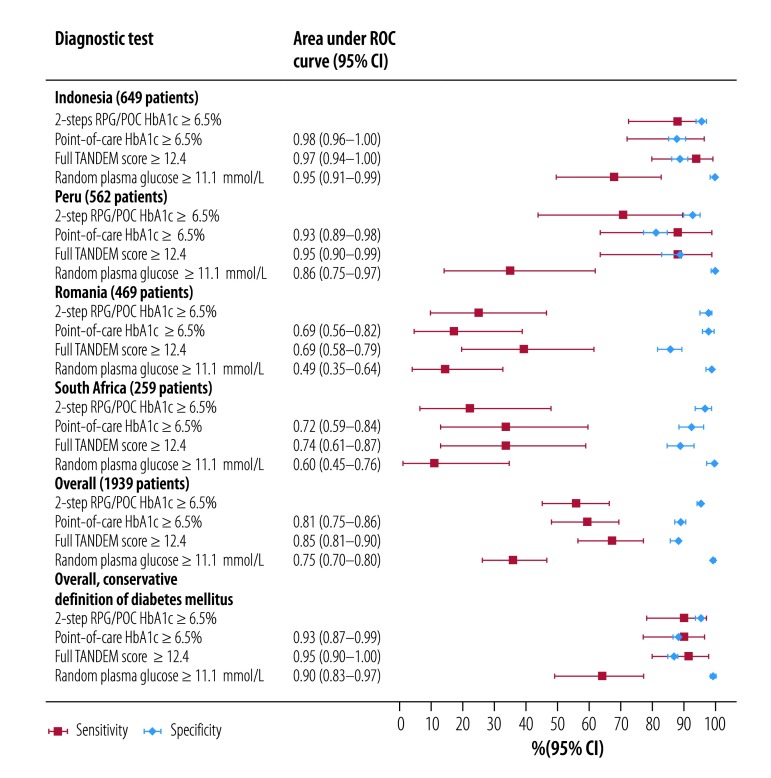
Diagnostic accuracy of diabetes mellitus markers in patients with newly diagnosed tuberculosis recruited to the Concurrent Tuberculosis and Diabetes Mellitus study, by country, 2014–2016

CI: confidence interval; HbA1C: glycated haemoglobin; ROC: receiver operating characteristic curve; RPG/POC; random plasma glucose point-of-care HbA1c test; TANDEM**:** Concurrent Tuberculosis and Diabetes Mellitus study.

Notes: In the two-step combination test, point-of-care HbA1c was included only if random plasma glucose was ≥ 6.1 mmol/L and ≤ 11.1 mmol/L. The full TANDEM score included age, point-of-care HbA1c and random plasma glucose. The standard definition of diabetes mellitus was laboratory-measured HbA1c ≥ 6.5%; conservative definition was HbA1c ≥ 7.0%.

Fig. 2 summarizes the accuracy of key diagnostic tests (details for each country are available from the repository^14^). All diabetes markers were more accurate in Indonesia and Peru, where patients newly diagnosed with diabetes had higher levels of HbA1c (Fig. 1). Sensitivity and specificity were lower in Romania and South Africa, where newly diagnosed patients had modest elevations of HbA1c. The two-step combination of achieved some of the highest combinations of sensitivity and specificity overall compared with other tests. For example, in Indonesia, the two-step combination had a sensitivity of 88.2% (95% CI: 72.5–96.7) and specificity of 96.0% (95% CI: 94.2–97.4). The sensitivity was higher (94.1%; 95% CI: 80.3–99.3) with a different lower point-of-care HbA1c cut-off (6.0%), without a substantial cost to specificity (91.1%; 95% CI: 88.5–93.2). In Romania, however, the respective sensitivity was much lower at 37.5% (95% CI: 18.8–59.4) and specificity was 87.8% (95% CI: 84.1–90.9), even using a point-of-care HbA1c cut-off of 6.0%. The full TANDEM score performed better in South Africa (0.74: 95% CI: 0.61‒0.87), relative to point-of-care HbA1c and the two-step combination. However, this option would be more expensive, requiring both random plasma glucose and point-of-care HbA1c tests as well as other risk markers. Sensitivity could possibly be further increased in Romania and South Africa with lower thresholds for each test, but the lower specificity would result in more expensive confirmatory tests.

Table 3 shows the combined diagnostic accuracy of individual diabetes markers, risk scores and published diabetes risk scores. The full TANDEM score achieved the highest area under the ROC curve overall (0.85; 95% CI: 0.81–0.90), followed by the restricted TANDEM score (0.84; 95% CI: 0.79–0.88), and a single point-of-care HbA1c measure (0.81; 95% CI: 0.75–0.86). The single random plasma glucose measure was also useful (0.77; 95% CI: 0.70–0.83). The three previously published diabetes risk scores had similar diagnostic accuracy as determined by the area under the ROC curve (0.70 to 0.72; 95% CI: 0.64–0.77). However, age alone was a stronger predictor of undiagnosed diabetes than these risks scores (0.75; 95% CI: 0.70–0.80). Weight-to-hip ratio and body mass index had poor diagnostic accuracy.

**Table 3:** Diagnostic accuracy of diabetes mellitus tests applied to applied to tuberculosis patients in the Concurrent Tuberculosis and Diabetes Mellitus study, 2014–2016

Diabetes mellitus marker and cut-off	Laboratory-measured HbA1c ≥ 6.5%^a^				Laboratory-measured HbA1c ≥ 7.0%^a^		
	Area under ROC curve	Sensitivity, % (95% CI)	Specificity, % (95%CI)		Area under ROC curve	Sensitivity, % (95% CI)	Specificity, % (95%CI)
**Single markers**							
Point-of-care HbA1c							
5.7%	0.81 (0.75–0.86)	81.3 (71.8–88.7)	56.2 (53.8–58.5)		0.93 (0.87–0.99)	91.7 (80.0–97.7)	55.6 (53.2–57.9)
6.2%		75.8 (65.7–84.2)	70.4 (68.2–72.6)			91.7 (80.0–97.5)	69.8 (67.7–71.9)
6.5%		59.3 (48.5–69.5)	88.7 (87.2–90.2)			89.6 (77.3–96.5)	88.4 (86.8–89.8)
Random plasma glucose							
≥ 5.3 mmol/L	0.77 (0.70–0.83)	83.5 (74.6–90.3)	40.0 (37.7–42.3)		0.90 (0.83–0.97)	94.0 (83.5–98.7)	39.7 (37.5–42.0)
≥ 6.9 mmol/L		62.9 (52.5–72.5)	83.3 (81.5–85.0)			88.0 (75.7–95.5)	82.8 (81.0–84.5)
≥ 11.1 mmol/L		36.1 (26.6–46.5)	99.3 (98.8–99.7)			64.0 (49.2–77.1)	99.2 (98.7–99.5)
Fasting blood glucose							
≥ 4.8 mmol/L	0.78 (0.70–0.85)	83.1 (71.0–91.6)	47.7 (44.6–50.9)		0.87 (0.76–0.99)	87.5 (67.6–97.3)	46.8 (43.7–49.9)
≥ 5.2 mmol/L		74.6.(61.6–85.0)	65.8 (62.8–68.8)			87.5 (67.6–97.3)	64.7 (61.7–67.2)
≥ 7.0 mmol/L		35.6 (23.6–49.1)	98.4 (97.3–99.1)			75.0 (53.3–90.2)	98.1 (97.1–98.9)
**Two-step combination test**^b^							
Random plasma glucose ≥ 6.1 mmol/L & point-of-care HbA1c ≥ 6.0%	NA	63.4 (52.8–73.2)	89.3 (87.8–90.7)		NA	90.0 (78.2–96.7)	88.8 (87.3–90.2)
Random plasma glucose ≥ 6.1 mmol/L & point-of-care HbA1c ≥ 6.5%	NA	55.9 (45.2-66.2)	95.4 (94.3–96.3)		NA	90.0 (78.2–96.7)	95.1 (94.0–96.1)
**Derived risk scores**							
Full TANDEM score^c^							
≥ 11.6	0.85 (0.81–0.90)	82.4 (73.0–89.6)	62.8 (60.5–65.1)		0.95 (0.90–1.00)	93.8 (82.8–98.7)	62.0 (59.7–64.3)
≥ 12.4		67.0 (56.4–76.5)	87.4 (85.7–88.9)			91.7 (80.0–97.7)	86.7 (85.1–88.3)
Restricted TANDEM score^d^							
≥ 3.1	0.84 (0.79–0.88)	85.6 (77.0–91.9)	58.4 (56.1–60.7)		0.94 (0.90–0.98)	96.0 (86.3–99.5)	57.5 (55.3–59.8)
≥ 3.5		71.1 (61.0–79.9)	77.2.(75.2-79.2)			92.0 (80.8–97.8)	76.6.(74.6–78.5)
**Published risk scores**							
Oman score^e^							
≥ 10	0.72 (0.66–0.77)	41.0 (31.3–51.3)	84.4 (82.7–86.1)		0.78 (0.72–0.84)	50.0 (35.5–64.5)	84.0 (82.3–85.6)
≥ 5		82.0 (73.1–89.0)	56.4 (54.1–58.7)			92.0 (80.8–97.8)	55.6 (53.4–57.9)
Indian risk score^e^							
≥ 10.6	0.72 (0.67–0.77)	90.0 (82.4–95.1)	43.0 (40.7–45.3)		0.76 (0.69–0.82)	92.0 (80.8–97.8)	42.2 (40.0–44.5)
≥ 21		70.0 (60.0–78.8)	62.8 (60.5–65.0)			76.0 (61.8–86.9)	62.0 (59.8–64.2)
							
Finnish diabetes risk score^e^							
≥ 2	0.70 (0.64–0.75)	76.8 (67.2–84.7)	56.5 (54.2–58.8)		0.75 (0.68–0.83)	84.0 (70.9–92.8)	55.8 (53.5–58.1)
≥ 9		17.2 (10.3–26.1)	95.3 (94.2–96.2)			26.0 (14.6–40.3)	95.2 (94.2–96.1)
**Other markers**							
Urine dipstick							
≥ trace glucose	0.66 (0.60–0.71)	37.8 (26.8–49.9)	92.5 (91.1–93.8)		0.74 (0.67–0.82)	54.3 (39.0–69.1)	92.5 (91.1–93.7)
Age							
≥ 38 years	0.75 (0.70–0.80)	81.0 (71.9–88.2)	58.2 (55.9–60.5)		0.79 (0.73–0.84)	90.0 (78.2–96.7)	57.4 (55.1–59.6)
≥ 45 years		72.0 (62.1–80.5)	72.3 (70.2–74.4)			82.0 (68.6–91.4)	71.4 (69.3–73.4)
Body mass index							
≥ 25 kg/m^2^	0.57 (0.51–0.63)	20.0 (12.7–29.2)	92.1 (90.8-93.3)		0.61 (0.52–0.70)	30.0 (17.9–44.6)	92.1 (90.8–93.3)
≥ 30 kg/m^2^		6.0 (2.2–12.6)	99.0 (98.4–99.4)			10.0 (3.3–21.8)	98.9 (98.4–99.3)
Weight-to-hip ratio							
≥ 0.77	0.59 (0.53–0.65)	96.0 (90.0–98.9)	13.3 (11.8–15.0)		0.62 (0.54–0.70)	98.0 (89.4–99.9)	13.2 (11.7–14.8)
Male ≥ 0.89; female ≥ 0.85		46.5 (36.4–56.8)	65.6 (63.4–67.8)			58.0 (43.2–71.8)	65.6 (63.4–67.8)
Male ≥ 0.90; female ≥ 0.80		47.5 (37.3–57.6)	58.8 (56.5–61.1)			60.0 (45.2–73.6)	59.0 (56.7–61.2)

CI: confidence interval; HbA1C: glycated haemoglobin; NA: not applicable; ROC: receiver operating characteristic; TANDEM**:** Concurrent Tuberculosis and Diabetes Mellitus study.

^a^ New cases of diabetes mellitus were defined using two cut-offs of laboratory-measured HbA1c: (i) ≥ 6.5% and (ii) ≥ 7.0%.

^b^ Random plasma glucose ≥ 11.1 mmol/L was deemed to be diabetes mellitus. If random plasma glucose was ≥ 6.1 mmol/L then a point-of-care HbA1c test was performed.

^c^ Full TANDEM score includes age, point-of-care HbA1c and random plasma glucose.

^d^ Restricted TANDEM score includes age, random plasma glucose, body mass index and physical activity.

^e^ Finnish diabetes risk score includes age, body mass index, waist circumference, current blood pressure medication, history of high blood glucose, physical activity and consumption of fruits and vegetables, and takes values 0–20 with a suggested cut-off ≥ 9.15.^16^ Indian risk score includes age, body mass index, waist circumference, family history of diabetes mellitus and physical activity, and takes values 0–42 with a suggested cut-off ≥ 21.^17^ Oman diabetes risk score includes age, waist circumference, sex, body mass index, hypertension and family history of diabetes mellitus, and takes values 0–25 with a suggested cut-off ≥ 10.^18^

The optimal diagnostic cut-offs for the full and restricted TANDEM scores were ≥ 12.4 and ≥ 3.5, respectively. The optimal diagnostic cut-off for a single point-of-care HbA1c measurement was ≥ 6.0%, which achieved higher sensitivity (75.8% versus 59.3%), but lower specificity (70.4% versus 88.7%) than the standard ≥ 6.5% cut-off. For random plasma glucose, the standard cut-off of ≥ 11.1 mmol/L achieved very high specificity (99.3%), but low sensitivity (36.1%). The optimal cut-off of ≥ 6.9 mmol/L had higher sensitivity (62.9%), but lower specificity (83.3%). The standard ≥ 7.0 mmol/L cut-off for fasting plasma glucose achieved high specificity (98.4%), but low sensitivity (35.6%), while the optimal cut-off for fasting plasma glucose was ≥ 5.2 mmol/L.

The two-step combination of random plasma glucose and point-of-care HbA1c (≥ 6.0%) achieved sensitivity of 63.4% and specificity of 89.3%, comparable accuracy to a single point-of-care HbA1c test ≥ 6.5%. The modest sensitivity was the result of combining four populations with heterogeneous distributions of HbA1c among those with newly diagnosed diabetes (Fig. 1).

All screening tests performed substantially better when diabetes status was classified at the end of tuberculosis treatment at 6 months (repeated HbA1c ≥ 6.5%). For example, using the two-step combination of random plasma glucose (≥ 6.1 mmol/L) and point-of-care HbA1c (≥ 6.5%), sensitivity increased to over 90% and specificity to 100% (available from the repository^14^). Similarly, with a conservative gold standard definition of diabetes (HbA1c ≥ 7.0%), the overall diagnostic accuracy of all diabetes markers increased considerably (Fig. 2; Table 3).

When diabetes was defined by fasting plasma glucose ≥ 7.0 mmol/L, diagnostic accuracy of all markers was lower than with HbA1c. However, the strongest diagnostic metrics were similar (data are available from the repository^14^).

## Discussion

Early diagnosis of diabetes mellitus in patients presenting with tuberculosis may lead to improved treatment outcomes and reduced mortality. Our study compared many potential diabetes screening procedures in patients diagnosed with tuberculosis, including point-of-care tests, and new and existing risk scores, in varied settings and patient ethnicities.

We found a significant prevalence of previously undiagnosed diabetes in newly diagnosed tuberculosis patients. While the screening tests were only performed in tuberculosis patients with no previous diabetes diagnosis, nearly twice as many patients with both tuberculosis and diabetes were identified simply by asking about previous diagnosis and medication. Over the four sites combined, derived risk scores, point-of-care HbA1c alone and a two-step combination of random plasma glucose and point-of-care HbA1c were the best-performing methods for diabetes screening. The two-step combination is appealing as point-of-care HbA1c testing is only required for those with random plasma glucose ≥ 6.1 mmol/L. In this study population, the combined test reduced the need for point-of-care HbA1c testing by 70%, potentially saving costs (Laurence Y, London School of Hygiene & Tropical Medicine, unpublished data, 2018).^21^ Further, the two steps can be completed in one clinic visit without prior fasting by patients, allowing rapid diagnosis. Fasting tests can be difficult to obtain in tuberculosis clinics. In this study the fasting plasma glucose was missing for approximately 45% of the patients for whom it was indicated (those with an initial random plasma glucose ≥ 6.1 mmol/L). In contrast, we were able to obtain an HbA1c measurement from about 95% of patients. In our study, health-care workers were also sometimes reluctant to ask patients to return in a state of fasting, as they felt that antituberculosis medication should ideally be taken with food to reduce nausea (and increase patient adherence). There was thus a strong preference among staff and patients for a screening strategy that could be completed rapidly, in a single visit, without prior fasting.

The three published scores performed poorly at detecting diabetes among tuberculosis patients. All three scores were derived in the general population and rely heavily on body mass index and waist circumference,^16^^–^^18^ which are often affected in tuberculosis patients. Median body mass index of the participants was below 19 kg/m^2^ in Indonesia and South Africa in this study, which may have contributed to the poor accuracy of the scores.

The risk scores we derived demonstrated strong diagnostic accuracy but they require validation in other populations. The restricted TANDEM score included body mass index with parameters calibrated to tuberculosis patients with lower body mass. Accuracy of the restricted TANDEM score was only slightly lower, and it may be useful in settings where point-of-care HbA1c testing is not possible.

There was considerable heterogeneity between study settings in the distribution of HbA1c in patients with undiagnosed diabetes. This heterogeneity affected the diagnostic accuracy for most screening tests and may reflect differences in disease prevalence or health-system issues (such as quality of diabetes services, health insurance coverage and access to tuberculosis services).^22^ Consequently, regional circumstances need to be taken into account when developing best local practice.

Due to pragmatic difficulties during the intensive phase of tuberculosis care, diabetes was defined consistently on a single HbA1c measurement in this study, though individuals with a repeated HbA1c measure below 6.5% were not included in the case definition. We also performed secondary analyses using a conservative gold-standard diabetes definition and based on diabetes status at the end of tuberculosis treatment (6 months after baseline). In both these analyses, the diagnostic accuracy of all diabetes markers was markedly improved, and the two-step combination of random plasma glucose and point-of-care HbA1c achieved both sensitivity and specificity > 90%.

In practice, the type of screening tests used are often dictated by local circumstances, such as the opportunity for patient follow-up, feasibility of fasting and costs associated with the tests.^11^ A cross-sectional study in India compared the diagnostic performance of HbA1c and fasting plasma glucose to oral glucose tolerance as the gold standard.^12^ The authors reported that HbA1c performed better than fasting plasma glucose among tuberculosis patients.^12^ This contrasts with the general population, in whom HbA1c tests may be less sensitive.^23^ WHO approved HbA1c testing for diagnosis of diabetes in 2011.^10^ However, few studies have used the method in tuberculosis patients,^24^^,^^25^ despite its practical benefits in requiring only a single non-fasting measurement and having less day-to-day and intra-day variation than blood glucose.^11^ Concerns have been raised about the accuracy of HbA1c testing in patients with anaemia. In particular, iron-deficiency anaemia may overestimate HbA1c,^26^ especially among people with normal or moderately raised HbA1c (< 6.5%),^27^ while haemolytic anaemia can underestimate HbA1c.^26^ In our study there was no difference in mean HbA1c between moderate, mild and non-anaemic tuberculosis patients,^14^ a finding supported by another cohort study from India.^28^ HbA1c appeared to be somewhat lower in those with severe anaemia, but with only 26 such individuals in our cohort, we lacked the statistical power to explore this further. A cautious approach to interpreting HbA1c in the presence of severe anaemia may be warranted.

In summary the two-step combination of random plasma glucose followed by point-of-care HbA1c testing if the random plasma glucose was above a set threshold appeared feasible and performed consistently well across sites. The combination performed especially well when based on diabetes confirmed at 6 months or using a conservative definition of diabetes. Most tests did not perform well in sites where many patients had borderline values of HbA1c, suggesting that mild elevations of any diabetes marker should be treated with caution at the beginning of tuberculosis treatment. Marginal hyperglycaemia may not be treated initially due to the potential for drug interactions, adverse events and possible impact on adherence to tuberculosis drugs.^22^ Such borderline diabetes patients should be offered repeat testing at the end of tuberculosis treatment, and ideally on a regular basis thereafter.
